# Dying Nations

**Published:** 1876-05

**Authors:** 


### DYING NATIONS.

Why do nations die? Cultivated Greece and all-conquering Rome; Vandal, and Goth, and Hun, and Moor, and Pole, and Turk, all dead or dying! Why? Murdered by nations more powerful? Swallowed by earthquakes? Swept away by pestilence and plague, or starved by pitiless famine? Not by any of these. Not by the lightning and the thunder; not by the tempest and the storm; not by poisoned air or volcanic fires did they die, and do they die! They perish by moral degredation; the legitimate results of gluttony, intemperance, and effeminacy. When a nation becomes rich, then there is leisure and the means of indulging in the appetites and passions of our nature which waste the body and wreck the mind. As with nations, so with families. Wealth takes away the wholesome stimulus of effort, idleness opens the flood-gates of passional indulgence, and the heir of millions dies heirless and poor, and both name and memory ingloriously rot!

If then, there is any truth and force in argument, each man owes it to himself, to his country, and more than all, to his Maker, to live a life of temperance, industry, and self-denial as to every animal gratification, and with these, having an eye to the glory of God, this nation of ours will live with increasing prosperity and renown, until with one foot on land and another on sea, the angel of eternity proclaims time is no longer!

